# Georgia State Dental Society

**Published:** 1872-12

**Authors:** M. S. Jobson, R. I. Hampton

**Affiliations:** Rec. Secretary.; Cor. Secretary.


### AMERICAN JOURNAL

OF

### DENTAL SCIENCE.

Vol. VI. THIRD SERIES--DECEMBER, 1872. No. 8.

### ARTICLE I.

### *Georgia State Dental Society.*

In accordance with an adjournment of April 8th, 1871, the Georgia State Dental Society met in the Hall of the Kimball House, Atlanta, Ga., April 3rd, 1872, at 10 o'clock, A. M. President, Dr. E. Parsons, of Savannah, in the chair.

After calling the roll, the regular order of business was suspended for the consideration of applications for membership. The following gentlemen, after a favorable report from the Executive Committee, were elected active members of the Society: Dr. Wm. Crenshaw, Atlanta; Dr. L. S. Morse, Forsyth; Dr. R. I. Hampton, Rome; Dr. Rosewald Thornton, Calhoun.

The rules of order were further suspended, and a resolution offered and adopted to appoint a Committee of three on Publication. Drs. Hampton, Carpenter and Ford were appointed by the Chair.

On motion, the Society adjourned until 2½ o'clock, P. M.

AFTERNOON SESSION.

Society met according to adjournment. Dr, E. Parsons, President, in the chair.

The report of the Executive Committee being favorable on the names of Drs. McElhaney, West Point; E. B. Marshall, Atlanta, and R. B. Adair, Gainesville; they were elected active members of the Society.

On motion all dentists present, not members of the Society, were invited to seats with this body during the session.

Minutes of last meeting being read and confirmed, the regular order of business was suspended in order to hear the opening address by the President.

On motion, the address was received and a vote of thanks tendered the President.

The Committee on Histology, Physiology and Dental Chemistry, not being present, the subject was passed over.

The Committee on Operative Dentistry made a verbal report of much importance, through their Chairman, Dr. F. Y. Clark, of Savannah.

On motion, the report was received and presented for discussion. Society then adjourned until 7½ o'clock, P. M.

NIGHT SESSION.

Society met according to adjournment. Dr. Parsons, President, in the chair.

The subject of Operative Dentistry was again taken up, and after quite an elaborate discussion by Drs. Clark, Allen, Ford, Hampton and others, was continued for further consideration to-morrow.

A resolution was offered and carried, that we have a clinic to-morrow, between the hours of 8 and 10 o'clock, A. M. Operators: Drs. Hampton, Thornton and Carpenter. The Society then adjourned until 10 o'clock, A. M., to-morrow.

SECOND DAY'S SESSION.

Society met according to adjournment. Dr. Parsons, President, in the chair.

Dr. Billups, Chairman of Committee on Mechanical Dentistry, made a verbal report which was received and ably discussed by most of the members present; after which Dr.

W. F. Tignor, of Columbus, and J. M. Lonquist, of Thomaston, were elected to active membership.

A resolution was offered and adopted, to accept the kind invitation for an excursion up the Air Line Rail Road, by members of the Society of Atlanta.

The Chairman of the Committee on Dental Education being absent, no report was made, bnt being very important the subject was opened for discussion.

Drs. Clark, Allen, Parsons and others spoke with much earnestness and effect upon the importance of a higher attainment of dental education, and in behalf of this very desirable object, that a resolution be adopted by this Society, recommending the subject to the Southern Association and the various State Societies.

Society adjourned until 2½ o'clock, P. M.

### AFTERNOON SESSION.

Society met according to adjournment. Dr. Parsons, President, in the chair.

The President, after vacating the chair, read a very able paper on Anæsthesia, which was discussed by most of the members present throughout the session.

Society adjourned until 7½ o'clock, P. M.

### NIGHT SESSION.

Society met according to adjournment. President, Dr. Parsons, in the chair.

Next business in order being election of officers, an election was held, which occupied the entire night session, with the following results:

*President.*—E. M. Allen, Marietta, Ga.

1*st Vice-President.*—A. C. Ford, Atlanta, Ga.

2*nd Vice-President.*—J. B. Murphy, Atlanta, Ga.

*Cor. Secretary.*—R. I. Hampton, Rome, Ga.

*Rec. Secretary.*—M. S. Jobson, Perry, Ga.

*Treasurer.*—H. A. Lowrance, Athens, Ga.

*Executive Committee.*—F. Y. Clark and E. Parsons, Savannah; J. P. H. Brown, Augusta; S. G. Holland, Thompson; E. S. Billups, Atlanta.

Society here adjourned to meet at 8½ A. M. to-morrow.

THIRD DAY'S SESSION.

Society met according to adjournment. President, Dr. Parsons, in the chair.

After the installation of the officers elect, the President, Dr. E. M. Allen, appointed the following committees:

*Physiology, Histology, Pathology, Chemistry and Therapeutics.*—Drs. E. Parsons, M. H. Thomas, and W. H. Burr.

*Operative Dentistry.*—Drs. J. P. H. Brown, R. I. Hampton and A. C. Ford.

*Mechanical Dentistry.*—Drs. L. D. Carpenter, L. S. Morse and J. M. Lonquist.

*Dental Education and Literature.*—Drs. F. Y. Clark, H. A. Lowrance and W. F. Tignor.

*Arrangements.*—Drs. W. F. Tignor, Saml. Hape and J. P. H. Brown.

*To Revise Constitution and By-Laws.*—Drs. E. Parsons, A. C. Ford, J. P. H. Brown, R. I. Hampton and G. P. Campbell.

The Society then adjourned to meet in Narcross on the excursion.

[After the adjournment the whole Society embarked on a Lightning Express up the Air Line Rail Road, and soon reached Narcross, a little growing city, about thirty or forty miles from Atlanta, where we were received and entertained in magnificent style by the proprietor of the Thrasher House.]

Society met at noon. President, Dr. Allen, in the chair.

After a favorable report from the Executive Committee, on the application of Dr. Geo. Patterson, he was elected an active member of the Society.

The following named gentlemen were appointed delegates to the Southern Dental Association: Drs. E. Parsons, R. I. Hampton, W. F. Tignor, G. W. McElhaney, R. B. Adair,

Geo. Patterson, L. D. Carpenter, M. H. Thomas, H. T. Campfield, L. S. Morse and W. T. Cole.

Drs. Lowrance and McElhaney were appointed to draft resolutions of thanks. Also, Drs. Clark and Thomas respecting honorary degrees from Dental Colleges.

Adjourned until 7½ o'clock, P. M.

[Here the Society was forced to an adjournment by the announcement of dinner, and after the subject being well discussed, the party retired to the hall, where we had a real good old-fashioned dance, but were compelled to stop very abruptly to take the Lightning Express, in order to reach Atlanta by Night.]

Society met at 7½ o'clock, P. M. President, Dr. E. M. Allen, in the Chair.

After transacting miscellaneous business the Society adjourned to meet in Columbus, Ga., on the first Wednesday of next April, at 10 o'clock, A. M.

